# Determining the Advantages, Costs, and Trade-Offs of a Novel Sodium Channel Mutation in the Copepod *Acartia hudsonica* to Paralytic Shellfish Toxins (PST)

**DOI:** 10.1371/journal.pone.0130097

**Published:** 2015-06-15

**Authors:** Michael Finiguerra, David E. Avery, Hans G. Dam

**Affiliations:** Department of Marine Sciences, University of Connecticut, Groton, Connecticut, United States of America; Indiana University, UNITED STATES

## Abstract

The marine copepod *Acartia hudsonica* was shown to be adapted to dinoflagellate prey, *Alexandrium fundyense*, which produce paralytic shellfish toxins (PST). Adaptation to PSTs in other organisms is caused by a mutation in the sodium channel. Recently, a mutation in the sodium channel in *A*. *hudsonica* was found. In this study, we rigorously tested for advantages, costs, and trade-offs associated with the mutant isoform of *A*. *hudsonica* under toxic and non-toxic conditions. We combined fitness with wild-type: mutant isoform ratio measurements on the same individual copepod to test our hypotheses. All *A*. *hudsonica* copepods express both the wild-type and mutant sodium channel isoforms, but in different proportions; some individuals express predominantly mutant (PMI) or wild-type isoforms (PWI), while most individuals express relatively equal amounts of each (EI). There was no consistent pattern of improved performance as a function of toxin dose for egg production rate (EPR), ingestion rate (I), and gross growth efficiency (GGE) for individuals in the PMI group relative to individuals in the PWI expression group. Neither was there any evidence to indicate a fitness benefit to the mutant isoform at intermediate toxin doses. No clear advantage under toxic conditions was associated with the mutation. Using a mixed-diet approach, there was also no observed relationship between individual wild-type: mutant isoform ratios and among expression groups, on both toxic and non-toxic diets, for eggs produced over three days. Lastly, expression of the mutant isoform did not mitigate the negative effects of the toxin. That is, the reductions in EPR from a toxic to non-toxic diet for copepods were independent of expression groups. Overall, the results did not support our hypotheses; the mutant sodium channel isoform does not appear to be related to adaptation to PST in *A*. *hudsonica*. Other potential mechanisms responsible for the adaptation are discussed.

## Introduction

Evolutionary adaptation to a stressor involves selection for phenotypes that confer a fitness advantage in the presence of the stressor. Stressors can include temperature, predation, resource availability, and environmental toxins. Adapted phenotypes may pay a fitness cost compared to susceptible phenotypes in the absence of the stressor, because resources allocated to adaptation detract from the overall fitness of an organism [1, 2]. For example, adaptation to the toxic metal cadmium in *Drosophila melanogaster* has clear fitness costs and advantages. Cadmium-resistant flies had higher fecundity in a cadmium contaminated environment, an advantage over susceptible flies, but lower fecundity in the absence of cadmium compared to susceptible flies, a cost [3]. Further, a specific phenotype (e.g., resistant) may experience a change in fitness across an environmental gradient, i.e. a trade-off; resistant flies suffered a decrease in fecundity going from an unpolluted to polluted environment. Understanding the advantages, costs, and trade-offs of adaptation is necessary for predicting how a population will respond to a stressor.

The stressors believed to be important in the ocean include micro-algae that produce toxins, such as dinoflagellates of the genus *Alexandrium*. The single-celled *Alexandrium* spp. produces a suite of neurotoxins that poison animals that consume it. The toxins include saxitoxin (STX) and related compounds (together termed paralytic shellfish toxins, PST) that bind to the extracellular pore region of voltage-gated sodium channels, which inhibits nerve transmission and muscle function [4–6]. Consumption of contaminated shellfish can lead to sickness or death in humans (paralytic shellfish poisoning, PSP) [7]. Importantly, toxic *Alexandrium* spp. can alter marine food webs [8], and disrupt populations of copepods [9]. Copepods are believed to be the most abundant metazoans in the oceans [10], and are integral to marine food webs [11], being the link between primary producers and upper trophic levels such as fish [12, 13]. Adaptation of copepods to a variety of global stressors, including toxic algae, is documented [14].

Populations of the ubiquitous coastal copepod species *Acartia hudsonica* that have been historically exposed to PST-producing *Alexandrium* spp. showed local adaption. In common garden experiments exposed populations had higher fitness-related parameters (e.g., ingestion, egg production, survival) when challenged with a diet that contained toxic *A*. *fundyense* than populations naïve to the toxic dinoflagellate-an advantage [15–17]. In contrast, on a non-toxic diet there was no difference in egg production and ingestion rate between naïve and exposed populations; there did not appear to be a cost to adaptation to PST [18]. The mechanism by which *A*. *hudsonica* may be adapted to the toxic algae was unknown.

Adaptation to PST in other organisms has been linked to mutations in the voltage-gated sodium channel. In marine bivalve clams a single point mutation in the toxin binding site region of the sodium channel lowered sensitivity to PST by over 100-fold. Clams that possessed the mutant sodium channels, as either homo- or heterozygous, had increased burrowing capacity and feeding rates under toxic conditions compared to individuals with the wild-type channels [19]. A similar mutation in the sodium channel was found in garter snakes that were resistant to newts that produce tetrodotoxin, another sodium-channel blocking neurotoxin. Further, resistance to sodium channel blockers was also documented in copepods. Parasitic copepods, *Lepeophtheirus salmonis*, that were resistant to sodium channel blocking pyrethroid insecticides where found to have mutations at similar locations to the garter snakes and clams. These mutations lowered sensitivity, presumably through reduced binding affinity [20]. For *Acartia hudsonica*, five discrete reproductive phenotypes linked to adaptation to PSTs suggested a simple molecular mechanism [21]. Indeed, two variant sodium channels, a wild-type and mutant, were identified for *A*. *hudsonica* [22]. All copepods analyzed were found to express both isoforms, but in varying proportions [23]. Importantly, the mutation in *A*. *hudsonica* differed markedly from those in clams and snakes; the mutation in the copepod did not affect the binding site nor reduce the sensitivity of the sodium channel to PST [22]. Instead, the mutation appeared to affect the intracellular inactivation gate of the sodium channel.

The voltage-gated sodium channel is a transmembrane pore through which sodium ions may pass from outside to inside the cell. When a nerve cell is stimulated the sodium channel activates, allowing a rapid influx of sodium ions into the cell, causing depolarization and an action potential. Immediately after, the intracellular inactivation gate occludes the channel from the inside, stopping the flow of sodium ions into the cell, allowing the cell to repolarize. It is the initial flow of ions into cell that is blocked when saxitoxin binds to the extracellular side of the channel [5]. The clam and snake mutations alter the binding site, inhibiting STX from binding and blocking the channels. The effect of the copepod’s mutation on the cellular inactivation gate of the sodium channel is less clear. In humans, sodium channels with similar mutations are “leaky”, i.e. these channels allow sodium ions to pass into the cell even when inactive. Such residual currents are considered deleterious to nerve and muscle function and lead to disease [24–28]. Treatments for diseases associated with leaky sodium channels in humans include low doses of sodium channel blockers [29], which operate in a similar cellular fashion to PSTs and reduce the effects of the leaky channels. If the mutation in *Acartia hudsonica* causes leaky sodium channels, then copepods that express the mutant isoform may be at an advantage in the presence of PSTs compared to individuals that express the wild-type isoform. Further, because of the purported leaky channel, copepods that predominantly express the mutant isoform may suffer an undocumented fitness cost in the absence of the toxin compared to wild-type expression, and suffer a trade-off across toxic and non-toxic environments. This possibility offers a potential novel mechanism of adaptation to PST.

We undertook this study to test the hypotheses that the novel sodium channel mutation confers a fitness advantage, incurs a fitness cost, and results in a fitness trade-off for *Acartia hudsonica* when they encounter prey that produce PSTs. We measured metrics of fitness (e.g., egg production, ingestion rate, gross growth efficiency) along with sodium channel isoform expression ratios on individual copepods, while controlling the diet that they were fed.

## Methods

### Culture of Algae and Copepods

Copepods for culturing were collected from Avery Point, Groton, CT, USA (Latitude: 41.31519 N, Longitude: 72.06352 W) during January 2010 using a 200 μm mesh size conical plankton net, equipped with a solid cod end, gently towed ~1–2m below the surface. No permits or permissions were needed to collect specimens from any of the field locations; copepods are not endangered or threatened. In the laboratory, approximately 300–400 male and female *Acartia hudsonica* were immediately separated and fed maximum rations (>600 μgC L^-1^, [30]) of a non-toxic diet (standard diet) that consisted of the diatom *Thalassiosira weissfloggi* and the green flagellate *Tetraselmis sp*. in equal carbon proportions. The standard diet has been routinely used to rear *A*. *hudsonica* cultures in our laboratory [15]. Animals and food were kept on a 12:12 hour light: dark cycle at 15°C. For each experiment, a cohort was established by placing adults (~300–500) above a 200 μm mesh barrier and allowing females to lay eggs for three to four days. Healthy fertilized adult female copepods produced from those eggs (cohort) were randomly chosen for experiments. Development time did not differ among experiments (data not shown), so the ages of females within each experiment and among experiments did not vary by more than four days; therefore, there was no age bias within and among experiments. At least twenty generations had elapsed from initial culturing before conducting experiments, which eliminated or minimized maternal effects [31].

Algae were maintained in triplicate semi-continuous cultures by dilution twice a week with F/2 nutrient medium [32] and maintained on a 12:12 hour light: dark cycle at 18°C. The toxic dinoflagellate *Alexandrium fundyense* was originally isolated from the Bay of Fundy, NB, Canada [15]; no toxic cells were collected directly from the field for any experiment. Toxicity was measure by HPLC [33] and expressed as pgSTX equivalents cell^-1^; conversion to *f*moles cell^-1^ can be made by multiplying pgSTX equivalents by a conversion factor of 2.54. The cultured toxic cells had a mean toxicity of 12.8 ± 1.1, 12.5 ± 2.6, and 12.0 ± 0.6 pgSTX equivalents cell^-1^ for the dose-dependent experiments, and 14.9 ± 2.3 pgSTX equivalents cell^-1^ for the experiment on reduction in egg production rate. Toxin content of cells for the mixed-diet experiment was not measured (see below for experimental details). Cell toxicity was measured by HPLC analysis following the procedure in Dam and Haley [33]. The non-toxic congener species *Alexandrium tamarense* originated from the NOAA marine fisheries laboratory in Milford, CT [15]. The toxic and non-toxic *Alexandrium* cells have similar equivalent spherical diameters (33 and 32μm, respectively) and carbon content (2.8 × 10^−3^ and 2.6 × 10^−3^ μgC cell^-1^, respectively). Three days prior to each experiment one replicate each of the toxic and non-toxic *Alexandrium* spp. cultures were transferred to 15°C to acclimate.

### Dose-Dependent Experiments

We used a toxicological approach to control the toxic cell concentration of the food suspension fed to experimental copepods. We mixed cells of toxic *Alexandrium fundyense* with cells of non-toxic *Alexandrium tamarense* while keeping total cell concentration constant at 200 cells mL^-1^ (equivalent to ~560 μgC L^-1^, which represented a maximum ration). The five treatments were: 200, 20, 2, 0.2 & 0 toxic *A*. *fundyense* cells mL^-1^, with the remaining cell difference being non-toxic *A*. *tamarense*. Three experiments were performed, on April 2 &15 and June 18, 2011. No statistical differences within treatment groups among experiments were found, so the three experiments were pooled together for the analyses presented here.

**Fig 1:**
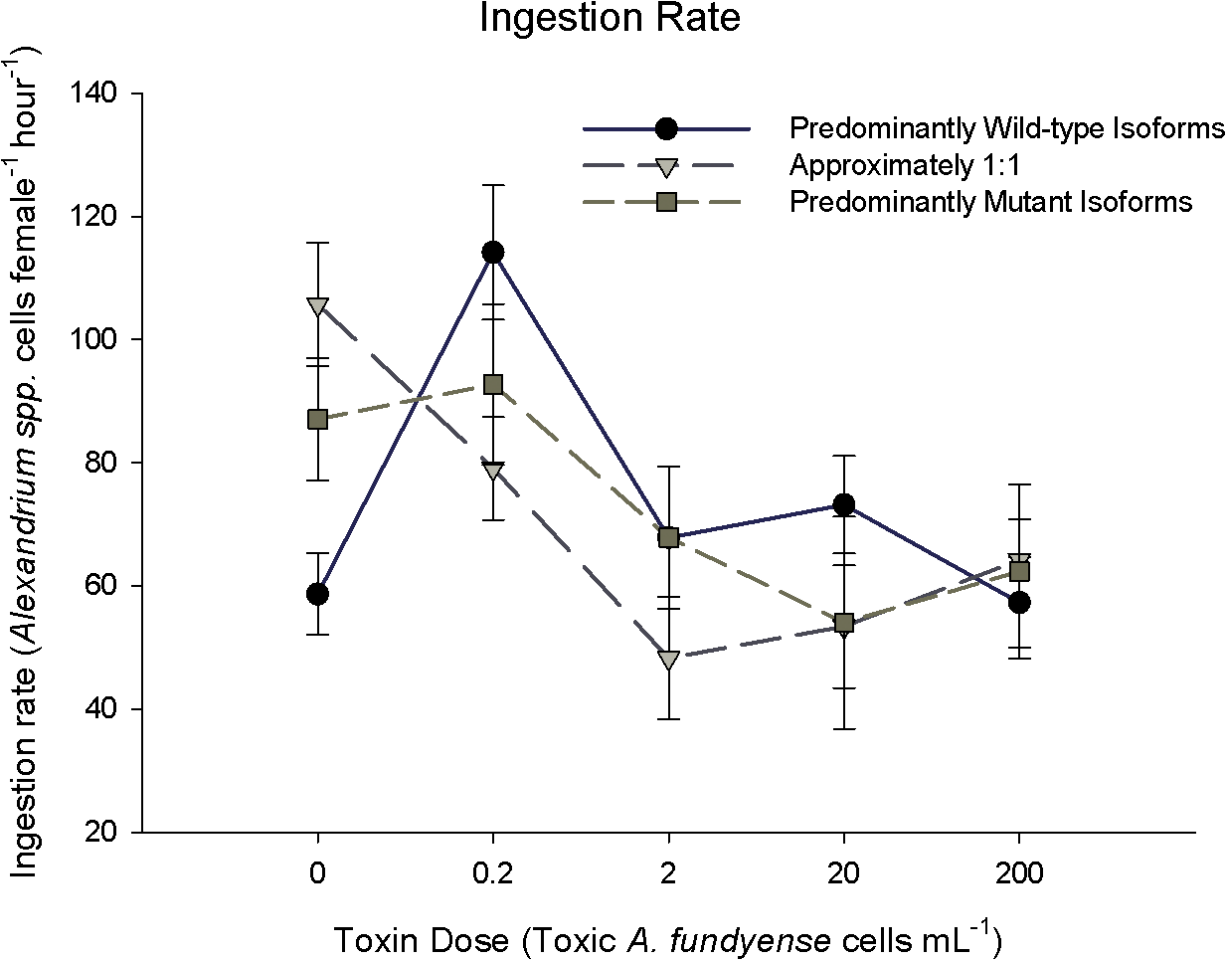
Reaction norms of individual ingestion rates versus toxic cell concentration, a proxy for PST dose. Points represent mean ± Standard Error. Symbols represent statistical equivalence within each food treatment only (*p*<0.05, ANOVA). Toxin dose is the concentration of toxic *A*. *fundyense* cells offered in diet. All diets contained a total of 200 *Alexandrium* spp. cells ml^-1^ (>550 μgC L^-1^; > non-limiting ration), with remaining proportion of cells being a non-toxic conger species *Alexandrium tamarense*. Expression groups were partitioned based on their wild-type: mutant isoform ratios (see Methods for detailed explanation). Sample sizes are as follows: 0 toxic cells: Wild-type (WT)-27, 1:1 12, Mutant (Mut): 13; 0.2 toxic cells: WT:11, 1:1 18, Mut: 7; 2.0 toxic cells: WT: 24, 1:1 12, Mut: 9; 20 toxic cells: WT: 19, 1:1 12, Mut: 5; 200 toxic cells: WT:22, 1:1 27, MUT:6. Conversion to *f*moles cell^-1^ can be made by multiplying pgSTX equivalents by a conversion factor of 2.54.

To measure ingestion rates, a single individual *A*. *hudsonica* female was sealed in a fully filled glass scintillation vials containing ~28mL of the appropriate food treatment (n = 24–30 per food treatment for each experiment), following the method of Senft et al, 2011 [34]. The clearance rate of *A*. *hudsonica* females at maximum ration is about 9 mL d^-1^ (derived from Fig 1 [18]). Therefore, even in the absence of cell growth, an individual *A*. *hudsonica* female would only clear about 1/3 of the food in experimental containers, not enough to bias ingestion rates [35]. Triplicate control vials, containing no copepods, at each food treatment were included. The copepod treatment and control vials were incubated at 15°C on a plankton wheel that rotated them end over end at 1.2 revolutions per minute for 24 hours. At the end of the incubation period, the contents of the vials with copepods were gently emptied into 100mm deep-sided Petri dishes. The copepod was removed and placed into 45 mL of the same food suspension in a 60 mm Petri dish to measure egg production (see below). The contents of the Petri dish from which the copepod was removed, which represents the ingestion assay, were then placed into Coulter-counter dilution vials and kept cool until cells were counted. Control vials were processed exactly as the copepod vials were. All samples were enumerated for total *Alexandrium* cells on a Beckman-Coulter Multisizer 4. Ingestion rates were calculated by cell disappearance based on five replicate initial cell concentration measurement vials [36]. Since toxic and non-toxic cells could not be distinguished with the particle counter, ingestion rates represent total cells ingested copepod^-1^ hour^-1^.

To measure egg production, copepods were transferred daily, for at least two days, to new Petri dishes containing a fresh suspension of the experimental food treatment. Eggs were counted daily. Gross growth efficiency (GGE) was calculated as the ratio of reproductive growth in the form of eggs to ingestion:

$$GGE\;=\;\frac{Carbon\;Growth}{Carbon\;Ingested}$$

In female copepods, it is often assumed that somatic growth ceases upon maturation (C6 molt) and excess energy is typically converted into egg biomass. This assumption relies on individual weight being at steady-state during the experiment [37]. In *Acartia* spp. weight changes during experimental incubations typically occurred under food-limited conditions [37], which was not the case here. Thus we assume egg production represents the energy equivalent of “adult growth”. Egg production and cell ingestion rates were converted to carbon using 45.7 ngC egg^-1^; [38], and 2.8 × 10^−3^ and 2.6 × 10^−3^ μgC cell^-^1 for *A*. *fundyense* and *A*. *tamarense*, respectively [15]).

At the end of the egg production experiment, copepods were immediately preserved to determine expression of each sodium channel isoform. Individuals were carefully captured by their antenna or urosome, to avoid crushing, with a pair of sharp-ended forceps (Dupont # 5 Tweezers), and immersed into a 1.5 mL microcentrifuge tube containing 200μl Tri Reagent (one copepod per tube; MRC, Inc., Cincinnati, OH 45212), and frozen at -80°C until molecular analyses (see below). While samples were typically processed immediately, there was no observed RNA degradation after one year of storage at -80°C [39]. Importantly, for the current experiments, within each food treatment we were able to calculate ingestion rate, egg production rate, GGE, and sodium channel isoform expression ratios for the same individual copepod within a food treatment (toxin dose).

### Mixed-Diet Egg Production Rate Experiments

To further test if the mutant isoform had any fitness consequences, copepods were fed one of two mixed-diets, each of which contained non-limiting rations of the standard diet (500 μgC L^-1^) supplemented with 200μgC L^-1^ (~70 cells mL^-1^) of either toxic *Alexandrium fundyense* or non-toxic *Alexandrium tamarense*. This approach eliminates any potential bias due to food-limitation; even if each copepod selected against all *Alexandrium* spp. cells there was enough food in the standard diet present to sustain maximum growth. Therefore, any differences seen between the toxic and non-toxic group are likely a result of the toxins, not food limitation. One hundred eighty adult female copepods per treatment were individually incubated in 45 mL of the food suspension in 60 mm Petri dishes and transferred daily to a fresh food suspension for three days, then preserved in Tri-Reagent as stated above. Mortality was less than 10% for both food treatments for the duration of the experiment (data not shown). Eggs from the three days of incubation for each individual were pooled and preserved with 4% acid Lugols solution in a 50 mL centrifuge tube. For counting, eggs were allowed to settle for one day, the upper portion of the tube was aspirated, and the remaining lower portion containing all eggs was rinsed into a clean Petri dish and counted.

### Change in Egg Production Rate Experiments

Lastly, we tested if the sodium channel mutant isoform mitigated the negative effects of toxins. Adult female *A*. *hudsonica* in three groups (n = 30–40 each group) were first individually fed non-limiting rations of the standard diet for two days. One group was then switched to a sole diet of non-limiting toxic *Alexandrium fundyense* (toxic group) while a second group was switched to a sole diet of non-limiting non-toxic *Alexandrium tamarense* (non-toxic group). The remaining group was maintained on the standard diet (standard diet group). Incubations continued for three more days (total: 5 day experiment), with all individuals preserved in Tri-Reagent as described above after the fifth day. Individual daily egg production rate for all five days was measured as described above. Change in EPR for each individual was calculated by subtracting the three-day average of the experimental diet from the initial two-day average on the standard diet.

### Molecular Analyses

An RNA-based approach was used to accurately determine the expression of each isoform in individual *A*. *hudsonica*. High quality RNA was extracted and reverse-transcribed according to the “Modified Zymo Method” [39]. Briefly, bead beating was used to homogenize individual copepods, and extraction was accomplished by coupling Zymo Direct-zol (Zymo Research, CA) reagents and phenol: chloroform purification steps. The first-strand cDNA was diluted three-fold with 10 mM Tris·HCl (pH 8) and stored at -20°C. Two SYBR green (Fast SYBR, Life Technologies)-based quantitative polymerase chain reactions (qPCR), one for each isoform (Primers- Mutant: AscComF3 & AscMuR1; Wild-type: AscComF3 & AscNorR2 [23]), were conducted for each individual copepod sample. These results were highly correlated to known isoform ratios and reproducible for the same sample; i.e. with no differences among replicate wells, or replicate runs of the same sample performed on different days. Expression artifacts from long handling times were minimized since all copepods were immediately preserved after the termination of each experiment. Importantly, this method could accurately detect the relative proportion of each sodium channel isoform, regardless of abundance, *in vivo*. As stated earlier, all copepods appear to express both isoforms, but in varying ratios. Thus, we used two metrics for our analyses. First, we treated ratios of wild-type: mutant isoforms as a continuous function and measurement value, comparing individual ratios among food treatment groups. The majority of copepods express both isoforms within a factor of 2–3 of each other, but there are extremes in either direction [23]. These extremes could bias analyses using individual ratios. Therefore, in addition to comparing wild-type: mutant ratios among treatments, we partitioned copepods into expression groups- those that predominantly expressed either mutant (PMI) or wild isoforms (PWI), and those that expressed both channel isoforms approximately equally. Further, discrete expression groups aided in analyses from a reaction norm perspective [40] The PMI and PWI expression groups were defined by isoform ratios that were greater than 2. To determine if these groupings biased results, we also tested other criteria for defining expression group (e.g., using a four-fold difference for isoform ratio), but this did not alter the results; therefore, we used the above definition for all analyses to increase sample size within each expression group.

### Statistical Analyses

Comparisons between less than two and greater than two expression groups were done with student’s t-test and a one-way ANOVA, respectively. For ANOVA tests, if differences were found (i.e. ANOVA p<0.05) then specific differences among groups were tested using Dunn’s or the Holm-Sidak method for non-parametric and parametric comparisons, respectively. Data for the mixed-diet experiment were not normally distributed, even after log-transformation (Shapiro-Wilk, p<0.05). Thus, a non-parametric test (Mann-Whitney U-test) was performed on the non-transformed data. For the dose-dependent experiments (I, EPR, GGE assays), a two-factor ANOVA was performed to determine if there was an interaction effect between toxic cell concentration and sodium channel expression groups. A sample size of eight (n = 8) per group was needed to detect differences among expression groups (power analysis, alpha = 0.05, power = 0.8; Sigma Plot software); this sample size was achieved for all experiments and experimental food treatments. Percentages (GGE) were arcsine-transformed prior to statistical analyses. The ingestion rate and GGE reaction norm data was not normally distributed (Shapiro-Wilk, p<0.05); an ANOVA on ranks (Kruskal-Wallis) was performed using original values with individual differences assessed using Dunn’s post-hoc test. For the experiment that tested change in EPR, a 1-sample t-test, using the null hypotheses of zero change, was used to determine if egg production (independent of expression group) changed due to diet. Causative relationships between egg production and expression groups were tested using ordinary linear regression. The relationship between I, EPR, and GGE as a function of toxin dose was determined using linear or polynomial regression of individual wild-type: mutant isoform ratios; best fit lines were assessed by the highest r^2^ and lowest p-values. All statistical tests were performed using Sigma Plot 11.0 with an alpha value of 0.05.

## Results

### Dose-Dependent Ingestion Rate, Egg Production Rate, and Gross Growth Efficiency

**Fig 2:**
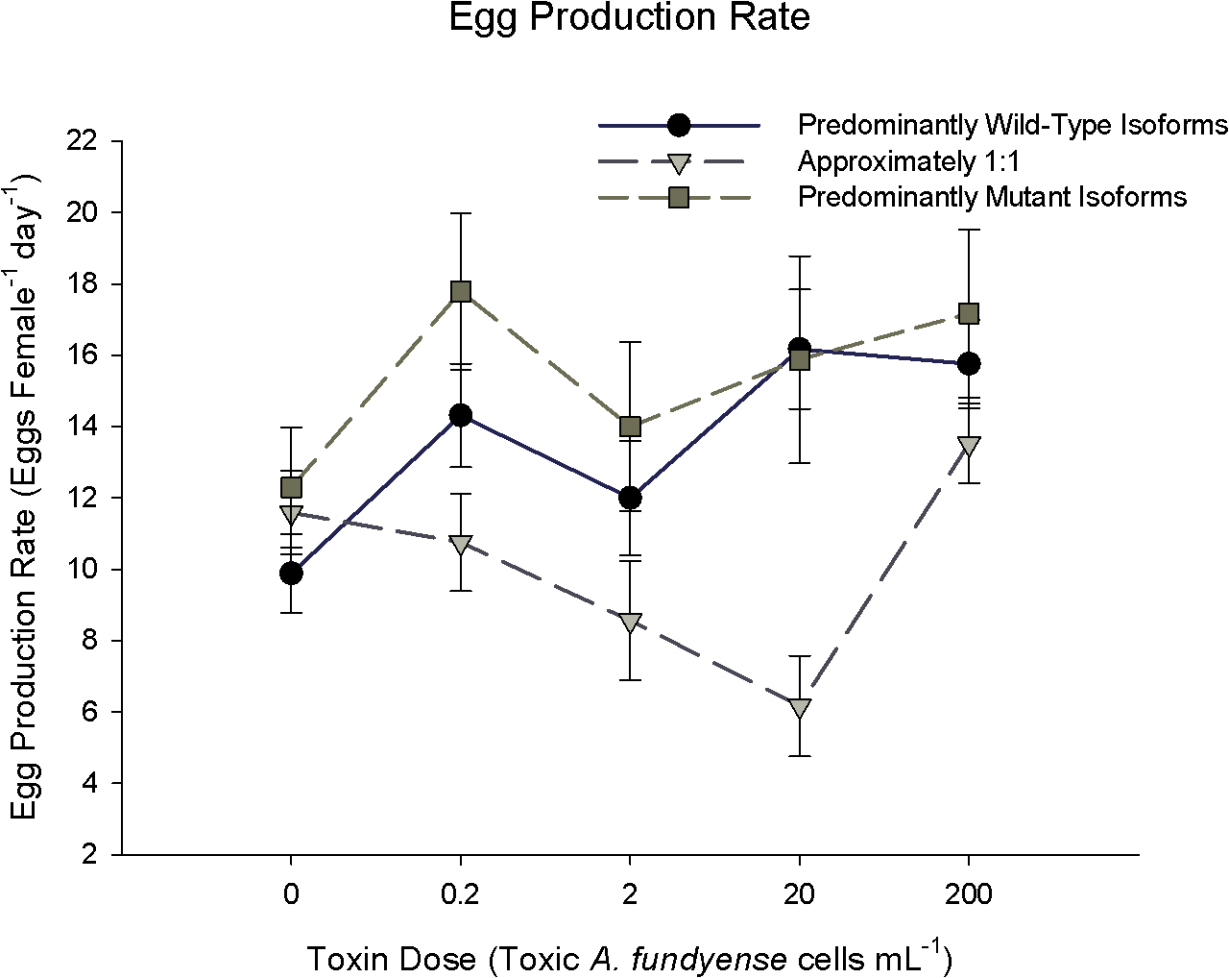
Reaction Norms of individual egg production rates versus toxic cell concentration. These are the same individuals from Fig 1. Points represent mean ± Standard Error. Symbols represent statistical equivalence within each food treatment only (*p*<0.05, ANOVA). Toxin dose is the concentration of toxic *A*. *fundyense* cells offered in diet. All diets contained a total of 200 *Alexandrium* spp. cells ml^-1^ (>550 μgC L^-1^; > non-limiting ration), with remaining proportion of cells being a non-toxic conger species *Alexandrium tamarense*. Expression groups were partitioned based on their wild-type: mutant isoform ratios (see Methods for detailed explanation). Sample sizes are the same as Fig 1. Conversion to *f*moles cell^-1^ can be made by multiplying pgSTX equivalents by a conversion factor of 2.54.

**Fig 3:**
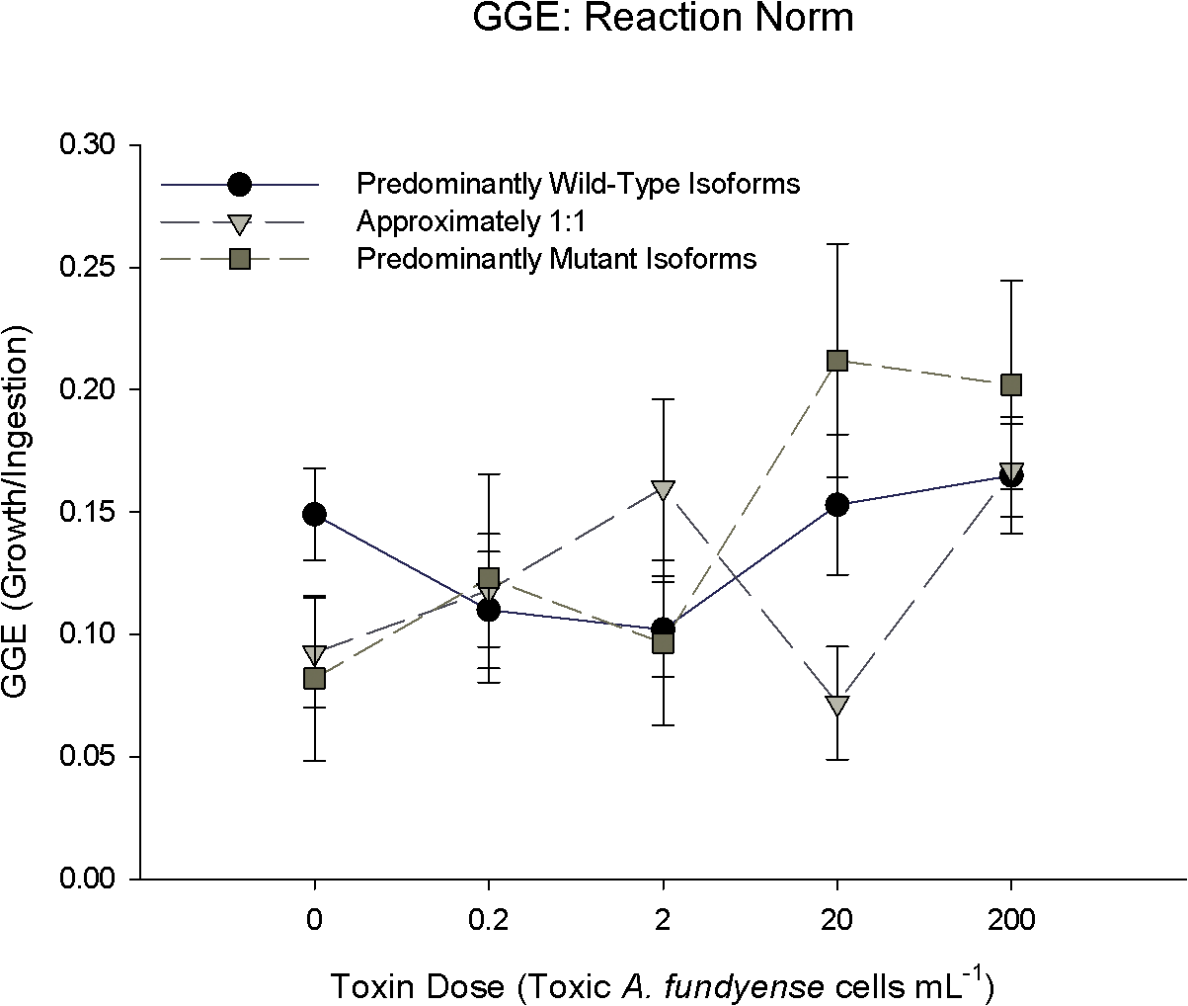
Reaction norm response of gross growth efficiency (GGE) versus toxic cell concentration. Data points are the same individuals from Figs 1 & 2 (for each food treatment). GGE was calculated from the carbon-scaled ratio of egg production rate to ingestion rate (see Methods for details). Other Fig legend details, including sample size, are the same as Figs 1 & 2.

**Fig 4:**
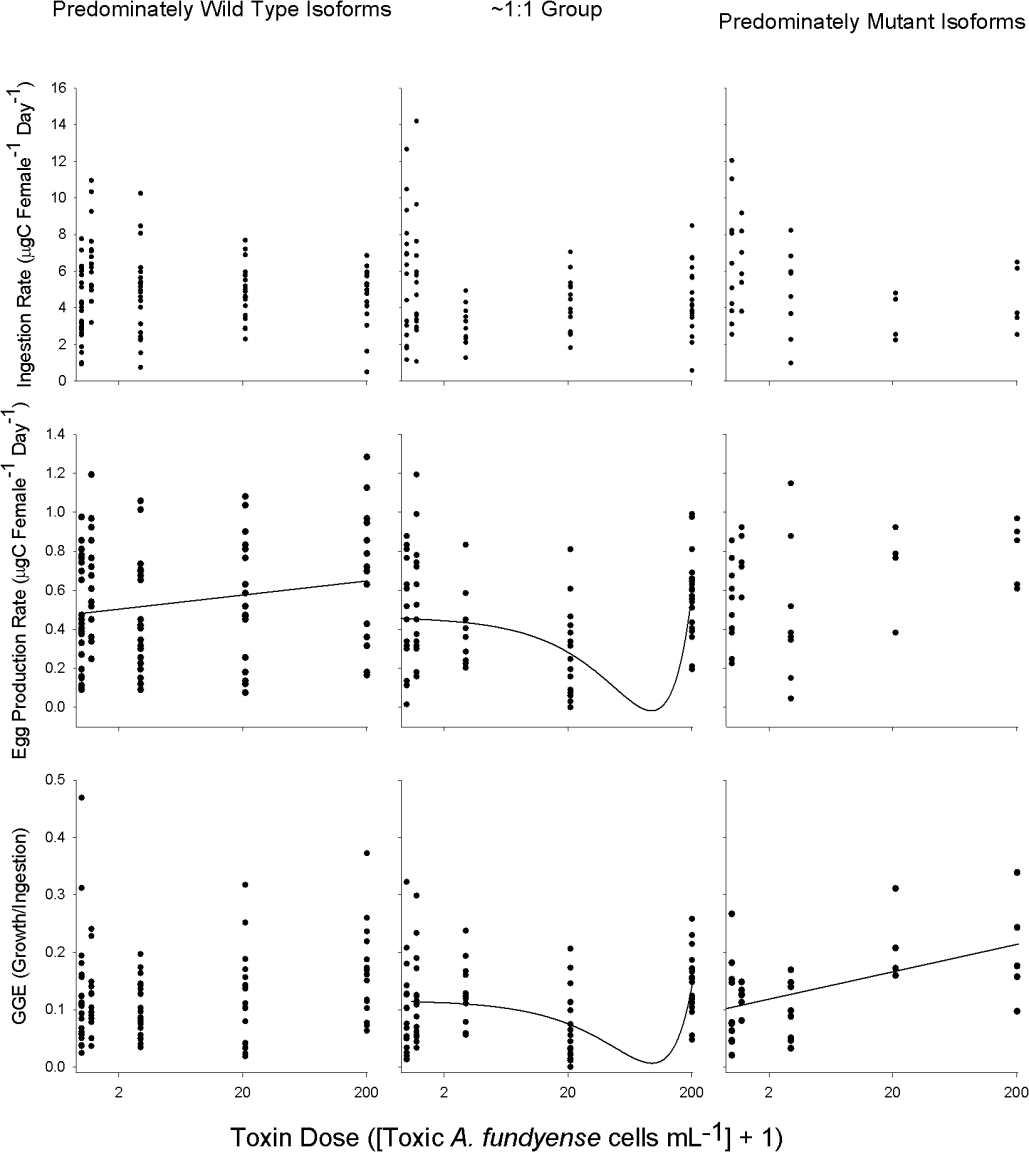
Individual wild-type: mutant isoform ratios from each expression group, with best fit lines, for the ingestion rate (I; top row), egg production rate, (EPR; middle row), and gross growth efficiency (GGE; bottom row) experiments. Dose is expressed as actual dose + 1 to aid in visualization of log_10_ scale. A line denotes a significant fit. The r^2^ values for the best fit lines are: PWI group EPR = 0.06; EI group EPR = 0.017, GGE = 0.09; PMI group GGE = 0.23. Equations for each best fit line can be found in results section. Sample sizes are the same as Figs 1–3.

The relationships between ingestion rate (Fig 1), egg production rate (Fig 2), and gross growth efficiency (Fig 3) and toxin dose as a function of sodium channel expression group, the reaction norms of ingestion, were complex. In the absence of toxins the PWI expression group showed a lower ingestion rate compared to the PMI and EI expression groups (Fig 1; Dunn’s Method p<0.05). At 0.2 toxic cells mL^-1^ the PWI expression group showed marginally higher ingestion (~20%) than the EI phenotype (Dunn’s Method, p<0.05); however, there were no significant differences between the PWI and PMI expression groups, and between the EI and PMI expression groups (Fig 1; 0.2 toxic cells mL^-1^; Dunn’s Method p<0.05). There was no relationship between ingestion rate for each expression group and toxin dose (Fig 4; ingestion rates, best-fit lines p>0.05). While there was no main effect on ingestion rate for expression group, there was an interaction effect between expression group and toxin dose (Table 1: Ingestion Rate; 2-factor ANOVA, p<0.05).

**Table 1:** Two-factor ANOVA tables for dose-dependent experiments, data same as Figs 1–3.

**Ingestion Rate**					
**Source of Variation**	**DF**	**SS**	**MS**	**F**	**P**
Toxic Treatment	4	32557	8139.3	6.831	<0.001
Expression Group	2	1069	534.7	0.449	0.639
Treat. × Exp. Group	8	36311	4538.8	3.809	<0.001
Residual	205	244251	1191.5		
Total 219	219	306187	1398.0		
**Egg Production Rate**					
**Source of Variation**	**DF**	**SS**	**MS**	**F**	**P**
Toxic Treatment	4	475.431	118.86	3.534	0.008
Expression Group	2	814.457	407.23	12.108	<0.001
Treat. × Exp. Group	8	581.373	72.67	2.161	0.032
Residual	196	6591.89	33.63		
Total	210	8573.63	40.83		
**Gross Growth Efficiency**					
**Source of Variation**	**DF**	**SS**	**MS**	**F**	**P**
Toxic Treatment	4	0.102	0.0256	2.814	0.027
Expression Group	2	0.012	0.0059	0.659	0.519
Treat. × Exp. Group	8	0.139	0.0174	1.916	0.060
Residual	192	1.745	0.0090		
Total	206	2.010	0.0097		

Abbreviations: Treat. = Toxic Treatment; Exp. Group = Expression Group. See methods for details concerning expression group definitions.

The egg production rate (EPR) reaction norm also showed no clear trend between the mutant sodium channel and toxicity. There was no difference in EPR among expression groups when zero toxic cells were present (Fig 2; ANOVA, p>0.05). At 0.2 toxic cells mL^-1^ EPR of the PMI expression group was approximately 20% greater than the PWI and EI expression groups (Fig 2; Holm-Sidak, p<0.05). Similarly, EPR in the PMI and PWI expression groups was two-fold greater than the EI expression group at 20 toxic cells mL^-1^ (Fig 2; Holm-Sidak, p<0.05). There was a positive, albeit weak, linear relationship between EPR and increasing toxin dose for the PWI expression group versus toxin dose (Fig 4 EPR: PWI; EPR = 0.5 + (0.00097 * Toxic Cells), r^2^ = 0.06, p<0.05). A quadratic equation was fit to the EI expression group (Fig 4 EPR: EI; polynomial order 2, EPR = 0.454 –(0.01 * Toxic Cells) + (0.00005 * Toxic Cells^2^), r^2^ = 0.17, *p* = 0.004) with a minimum close to 200 toxic cells mL^-1^. There was no relationship between EPR and toxic cell concentration for the PMI group (Fig 4 EPR: PMI; best fit lines p>0.05). There was a main effect on EPR for both expression groups and toxin dose, as well as an interaction effect between the two factors (Table 1; Egg Production Rate; 2-factor ANOVA, p<0.05).

Since ingestion and egg production rate were measured for each copepod, gross growth efficiency (GGE) was calculated and compared among expression groups as a function of toxin dose (Fig 3). At zero toxic cells the PWI expression group had a higher GGE, by approximately 50%, compared to the EI and PMI groups (Dunn’s Method, p<0.05), which suggests a cost to the mutant isoform. There was no difference in GGE among expression groups at 0.2, and 200 toxic cells mL^-1^ (Dunn’s Method, p>0.05). At two toxic cells mL^-1^ GGE for the EI expression group was higher than the PWI and PMI (Dunn’s Method, p<0.05). The PMI group had higher GGE than the EI expression group at 20 toxic cells mL^-1^(Dunn’s Method p<0.05), but the PWI group was equivalent to the other two expression groups (Kruskal-Wallis ANOVA on Ranks p>0.05). There was no relationship between GGE and toxic cell concentration for the PWI expression group (Fig 4; GGE, no best fit: p>0.05). A quadratic equation was used to describe the relationship between GGE and toxin dose for the EI expression group (Fig 4, polynomial second order; GGE = 0.117 –(0.07 * Toxic Cells) + (0.04 * Toxic Cells^2^), r^2^ = 0.09; *p* = 0.048) with a minimum value close to 200 toxic cells mL^-1^, while the PMI expression group was described by a positive linear relationship (linear regression, GGE = 0.104 + (0.05 * Toxic Cells), r^2^ = 0.23, *p* = 0.002). There was a main effect of toxin dose on GGE, and an interaction effect between expression group and toxin dose for GGE (Table 1; Gross Growth Efficiency, 2-factor ANOVA, p<0.05).

**Fig 5:**
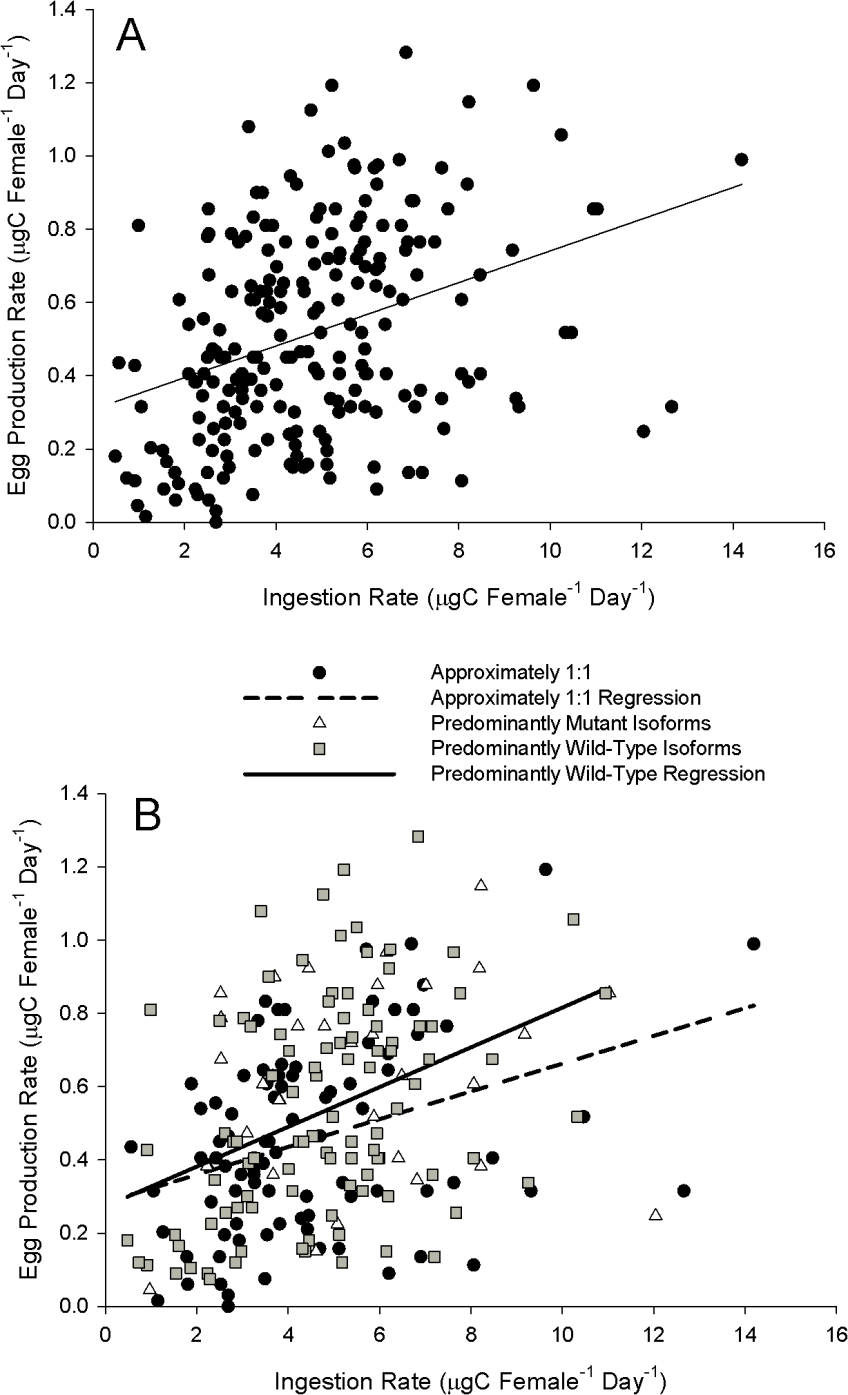
Egg production rate as a function of ingestion rate (measurements were taken on the same individual, which correspond to Figs 1 & 2). A) All individuals plotted together, independent of isoform ratio and toxin dose. Linear regression *p*<0.001; EPR = 0.308 + (0.0433 * Ingestion Rate); r^2^ = 0.13; n = 220. B) The same data, but partitioned according to isoform expression group (see methods for details). Linear regression *p*<0.05; predominantly wild-type (PWI) expression group: EPR = 0.273 + (0.0543 * Ingestion Rate), r^2^ = 0.15, n = 99; Approximately 1:1 (EI) expression group: EPR = 0.284 + (0.0379 * Ingestion Rate), r^2^ = 0.13, n = 88; predominantly mutant: no significant regression, n = 33.

Overall, combining all toxin doses and expression groups, there was a positive linear relationship between EPR and I (Fig 5A: linear regression; EPR = 0.308 + (0.0433 * Ingestion Rate); r^2^ = 0.13, *p*<0.001). This positive relationship was also found for the individual PWI and EI expression groups (Fig 5B: Linear regression; PWI expression group: EPR = 0.273 + (0.0543 * Ingestion Rate); r^2^ = 0.15; *p*<0.05; EI expression group. EPR = 0.284 + (0.0379 * Ingestion Rate); r^2^ = 0.13, *p*<0.05), but not for PMI expression group (linear regression, p>0.05).

### Mixed-Diet Egg Production

**Fig 6:**
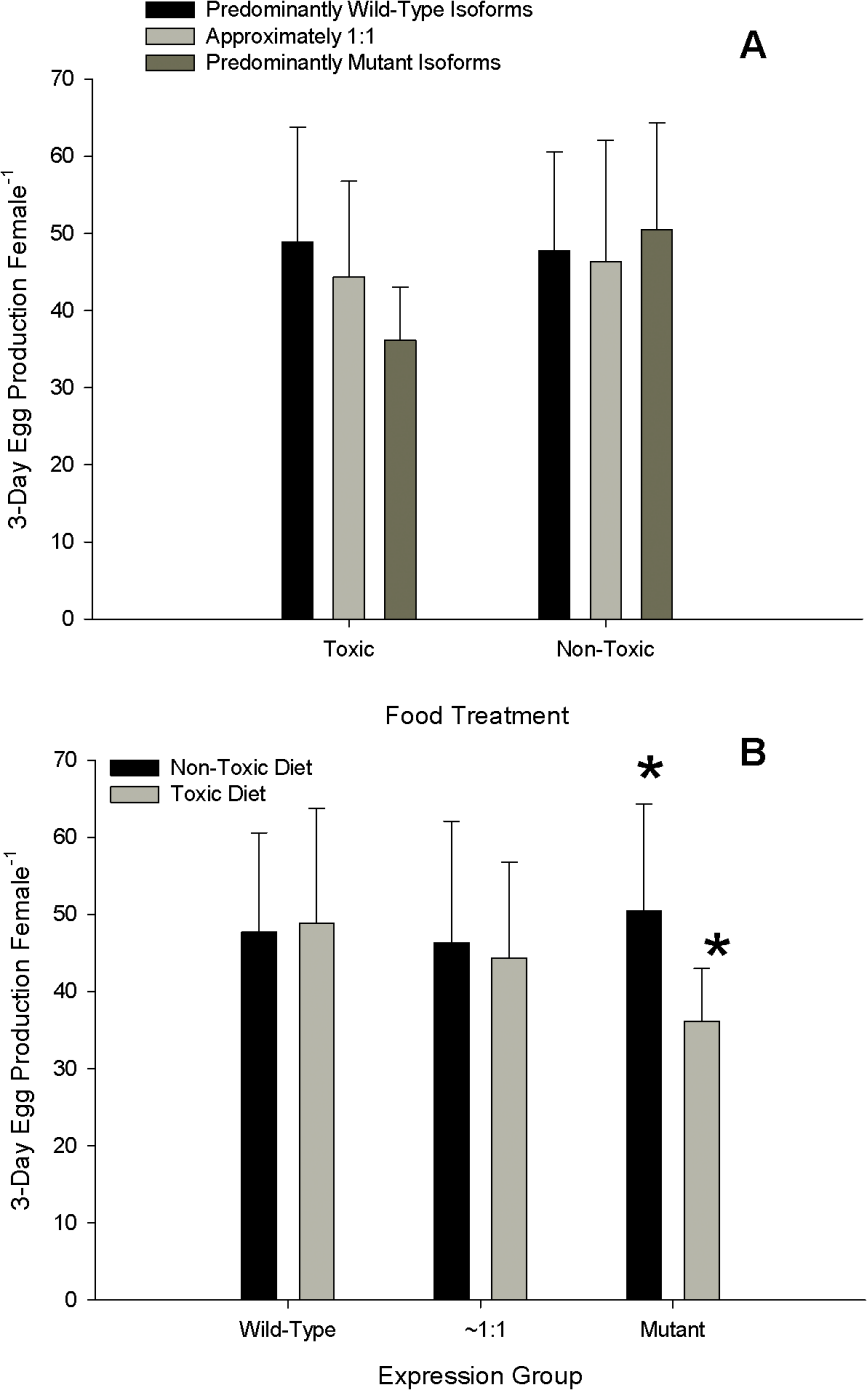
Three-day egg production on mixed-diets containing toxic or nontoxic food. Each diet consisted of 500μgC L^-1^ of the standard diet (equal carbon of *Tetraselmis sp*. and *Thalassiosira weissfloggi*) mixed with 200 μgC L^-1^ of either toxic *Alexandrium fundyense* or non-toxic *Alexandrium tamarense*. The data are plotted as A) within toxic and non-toxic treatments among expression groups (n = 164), and B) within expression groups between food treatments (n = 165). Abbreviations- Wild-type: Predominantly wild-type isoform expression (PWI); ~1:1: approximately equal isoform expression (EI); Mutant: predominantly mutant isoform expression (PMI). Symbol in B represents statistical differences between EPR on toxic and non-toxic food for the PMI expression group (student’s t-test, *p*<0.05). Conversion to *f*moles cell^-1^ can be made by multiplying pgSTX equivalents by a conversion factor of 2.54.

**Fig 7:**
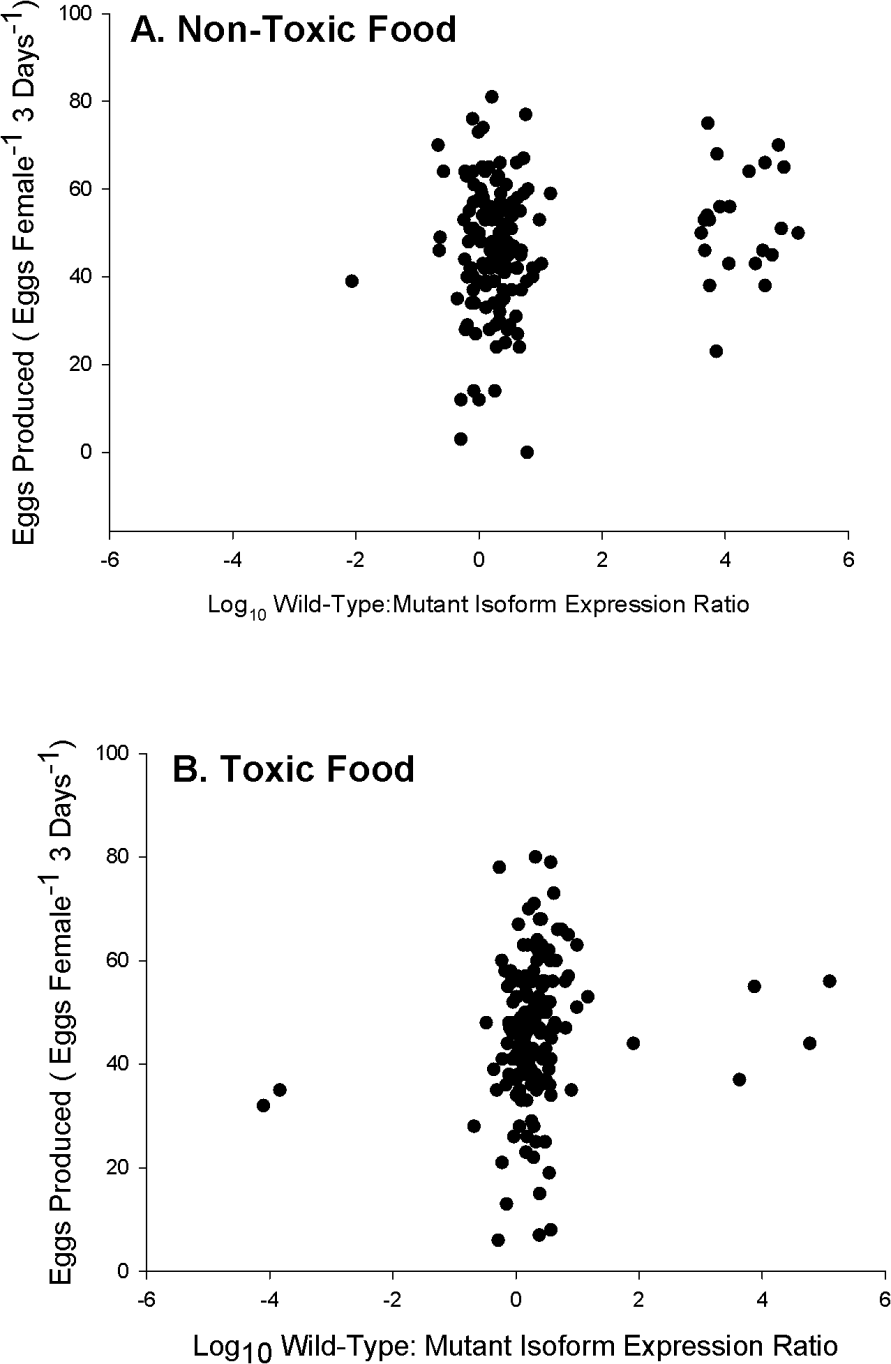
Three-day total egg production (same data as Fig 6) as a function of the log_10_ ratio of wild-type: mutant sodium channel isoform expression in the A) non-toxic and B) toxic food treatments. No relationships were found (linear regression, *p*>0.05).

There were no differences among expression groups for both toxic and non-toxic diets (Fig 6A). The PWI and EI expression groups showed no change in eggs produced between the non-toxic and toxic environments; however, the PMI expression group saw a reduction in eggs produced on the toxic diet (Fig 6B). There was no relationship between the ratios of wild-type: mutant isoforms and egg production; individuals with a higher proportion of the mutant isoform expression did not show increased egg production on a toxic diet, or lower egg production on a non-toxic diet (Fig 7, linear regression *p*>0.05). Food treatment and expression group had no main effect on eggs produced, and no interaction effect between food treatment and expression group for eggs produced on the mixed-diet was found (two-factor ANOVA, p>0.05).

### Reduction in EPR

**Fig 8:**
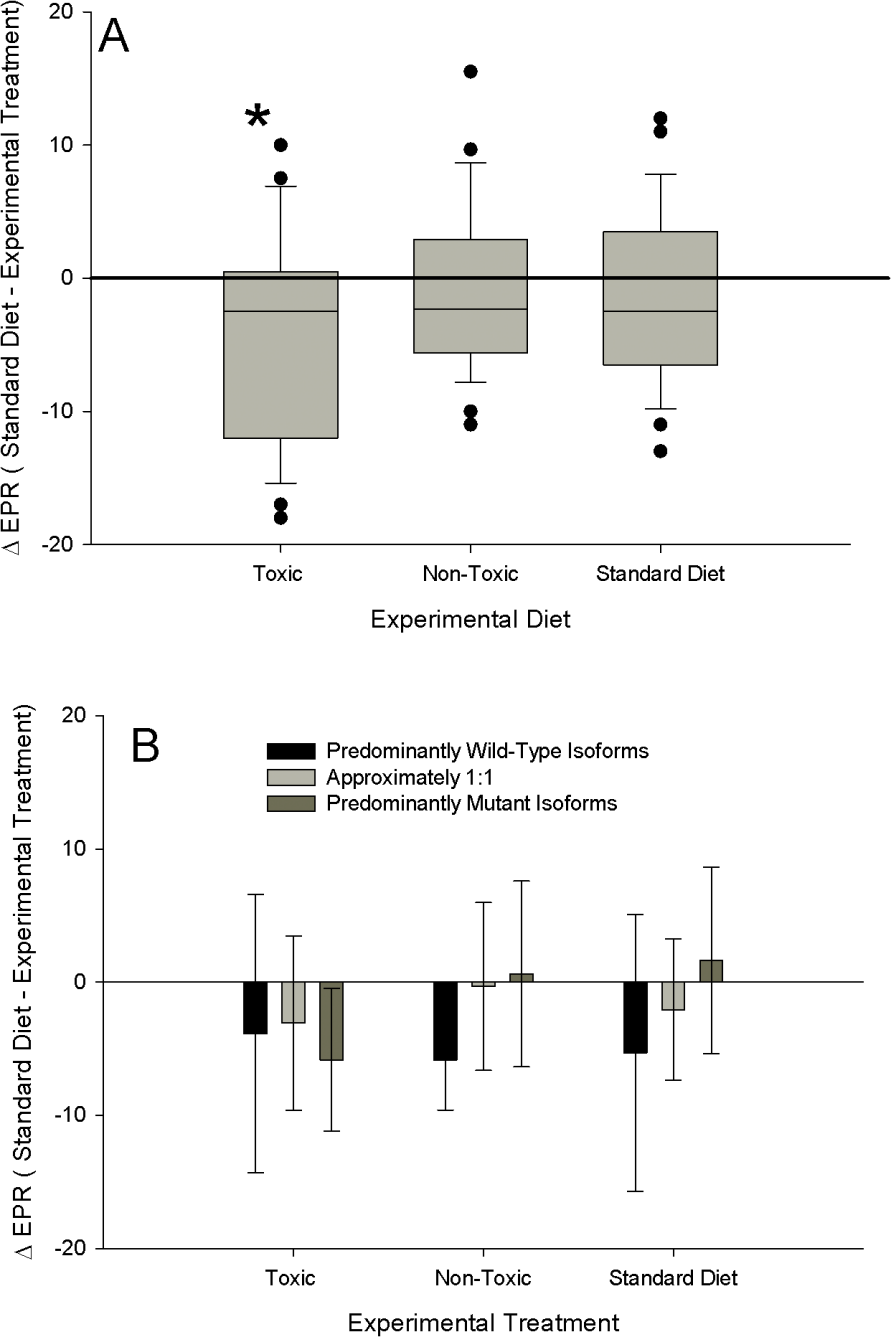
Individual changes in egg production rate for copepods that were initially fed the standard diet (500μgC L^-1^; equal C of *Tetraselmis sp*. and *Thalassiosira weissfloggi*) for two days then switched to either toxic *Alexandrium fundyense* (Toxic), non-toxic *Alexandrium tamarense* (non-toxic) or maintained on the standard diet (Standard Diet) for three more days. A) All data pooled together for each food treatment. The * indicates that only the Toxic treatment had a reduced EPR (1 sample t-test, H_o_ = no change); Sample sizes: toxic diet: 23, non-toxic: 29, standard diet: 27 B) Data viewed according to change in EPR for each expression group. There was no difference among groups within each food treatment and among food treatments within each expression group (all date points; ANOVA, *p*>0.05); Sample sizes: Toxic: wild-type (WT): 8, 1:1 10, mutant (Mut): 5; Non-Toxic: WT:5, 1:1 20, Mut:4; Standard Diet: WT:6, 1:1 13, Mut:8. For A: Line within the box is the median; perimeter lines and whiskers are 25^th^ & 75^th^ and 10^th^ & 90^th^ percentiles, respectively, and solid data points are outliers. Conversion to *f*moles cell^-1^ can be made by multiplying pgSTX equivalents by a conversion factor of 2.54.

The magnitude of the fitness penalty from a non-toxic to toxic environment was not associated with the sodium channel expression groups. Independent of expression groups, i.e. pooling all individuals within each food treatment, there was a reduction in EPR on toxic *A*. *fundyense*, but no reduction on the standard diet and non-toxic *A*. *tamarense* treatments, compared to the initial standard diet (Fig 8A; 1-sample t-test, H_o_ = zero change; toxic treatment *p*<0.05, non-toxic and standard diet treatments *p*>0.05). There was no difference among expression groups within each experimental diet, nor was there any difference within expression groups among diets (Fig 8B; ANOVA p>0.05).

## Discussion

Evidence has shown that historical exposure to toxic *Alexandrium* spp. resulted in local adaptation of *Acartia hudsonica* copepod populations [15, 17, 18]. The mechanism by which this adaptation was accomplished was not tested in those studies, but others have linked similar adaptation to neurotoxic prey to mutations in the toxin binding site of the sodium channel of the predators [19, 41]. A sodium channel gene of *A*. *hudsonica* has recently been sequenced and a mutation found [22]. While this mutation is not in the binding site of STX, it did show some characteristics that we hypothesized could enhance fitness when copepods feed on the toxic alga. Here, we rigorously tested whether or not the mutant sodium channel isoform conferred an advantage, cost, or trade-off to *A*. *hudsonica*. We also report, to our knowledge, the first comprehensive data set of ingestion rate (I), egg production rate (EPR), gross growth efficiency (GGE), and gene expression for the same individual copepod. Overall, our hypotheses were not supported. While some results suggest that the mutation influenced the phenotypic response, there was no consistent evidence of an advantage, cost, or trade-off of the mutant sodium channel isoform to PSTs. Other mechanisms that contribute to PST adaptation in *A*. *hudsonica* must be present.

### Advantages

An advantage of the mutant sodium channel isoform to PSTs is tested under toxic conditions and could be manifested in several ways. Broadly, an advantage in *Acartia hudsonica* would be evident if copepods that predominantly expressed mutant sodium channel isoforms always performed better under toxic food conditions compared to those that expressed wild-type isoforms. Adapted northern exposed populations of *A*. *hudsonica* had EPR and I rates that were ~200% greater than susceptible naïve populations [18]. A similar relative change in EPR between the putative tolerant (PMI, EI) and the susceptible (PWI) expression groups was not observed (Figs 2–3). PMI individuals only had the highest EPR at 0.2 toxic cells mL^-1^ (Fig 2). When other differences in ingestion, EPR, and GGE were observed among expression groups, the PMI and PWI groups were the same; thus, performance could not be attributed to the mutant isoform. Further, expression of the mutant isoform did not result in a lower-magnitude reduction of EPR from non-toxic to toxic food (Fig 8). There was also no relationship between EPR and individual wild-type: mutant isoform ratios on a toxic diet (Fig 7B). There was no consistent evidence across experiments to support this hypothesis.

Any differences in EPR, GGE, and I rate between tolerant and susceptible individuals should become larger with increasing toxin dose [42, 43]. This trend was also not observed. Increasing toxin dose did not influence the magnitude of fitness differences, if observed, among expression groups. For example, the largest difference in EPR (Fig 2) and GGE (Fig 3) among expression groups was observed at 20 toxic cells mL^-1^, not 200 toxic cells mL^-1^. Soft shell clams that are heterozygous for the PST resistant allele have a resistance in between the homozygous genotypes [44]. Even if we treat the EI group as intermediate between the PMI and PWI, there was no consistent evidence to support the prediction that fitness differences should increase with toxin dose. Copepods that expressed the mutant isoform did not have an increasing advantage at higher toxic cell concentrations.

Alternatively, an advantage of the mutant isoform may only come at a low or intermediate toxin dose. This assumes that the mutant isoform in *Acartia hudsonica* is similar to “leaky” sodium channels in humans [45], i.e. a malfunctioning intracellular inactivation gate that allows residual currents. In humans, small doses of sodium channel blockers are used to alleviate the symptoms of malfunctioning leaky sodium channels [29]. The channel blocking drugs bind to the channel and reduce residual currents. Since the mutation present in copepods does not affect the extracellular binding site, sodium channel blockers will bind to all sodium channel isoforms, wild-type and mutant, equally. By blocking a fraction of all sodium channels, the negative consequences of the leaky mutant isoforms are mitigated, and the overall function of the cell is returned to near normal. The dose, however, is critical. If the dose is too high, then too many sodium channels overall are blocked and the cell becomes impaired, regardless of isoform composition. Therefore, only a low dose of sodium channel blockers (e.g., low toxic *A*. *fundyense* cell concentration) may restore the *A*. *hudsonica* cells that predominantly express mutant sodium channels to near normal function. Thus, PMI individuals may have an advantage over PWI copepods under certain low-dose toxin environments. Since the EI group has proportionally fewer mutant isoform channels, it should require a lower dose than the PMI group to achieve this advantage. The PWI group, though, should always experience a decrease in fitness with increasing toxicity. Overall, there is little evidence to support this intermediate dose hypothesis.

An advantage at an intermediate dose for the PMI group over the EI and PWI groups was only seen for EPR at 0.2 toxic cells mL^-1^ (Fig 2). Toxic *Alexandrium* spp. is typically a small fraction of the overall phytoplankton biomass when present in the Gulf of Maine [46]. An increase in EPR at low toxic cell concentrations could select for individuals during low density toxic *Alexandrium* spp. blooms; however, there were no differences between PWI and PMI groups at any toxin dose for ingestion rate (Fig 1) and GGE (Fig 3). The mixed-diet experiments also corresponded to a low dose of PSTs, but again there were no differences among expression groups (Fig 6). Lastly, optimum performance values were never observed at intermediate doses for individual expression groups, nor were they different among groups (Fig 4). In fact, if a non-linear regression was significantly the best fit, it resulted in a minimum at an intermediate dose. Overall, differences among individual fitness parameters cannot be attributed to an advantage of the mutant isoform at intermediate doses.

The apparent increase in EPR (Figs 2 & 4: middle row) and GGE (Figs 3 & 4: bottom row) at higher toxin doses for some expression groups is contrary to previous reports [18]. Toxins produced by *Alexandrium* spp. are generally thought to be sub-lethal to copepods, instead acting to incapacitate copepods physiologically [47], leading to death by starvation [21]. If the toxins slow the nerves and muscles of the gut, the likely result might be a reduction in peristalsis required to move food through the digestive tract, potentially increasing the amount of time nutrients can be extracted from food. Since EPR was measured over numerous days, this could explain the higher EPR and GGE under more toxic conditions, but requires further experimentation to verify. It is also important to highlight some differences between our work and that of Colin and Dam [15, 18] that may contribute to the apparent inconsistencies. Data from Colin and Dam are from pooled individuals. The current study made all measurements on individuals. Further, our experiments tracked fitness measurements over the course of three to four days, while Colin and Dam typically only used 24 hour incubations for all measurements. These differences preclude precise comparisons.

Negative selection against toxic cells due to copepod behavior could have also biased the results. Complete avoidance of toxic cells is unlikely; regardless of the proportion of toxic to non-toxic cells offered they will still consume some toxic cells [15, 48–50]. For example, cell avoidance cannot explain the reduced ingestion rates of copepods offered mixed-proportions of toxic and non-toxic *Alexandrium* spp. cells [16]. If total avoidance occurred, as opposed to physiological incapacitation from consumption of toxic cells [47], then ingestion rate should have remained constant through time, but dependent on the total concentration of non-toxic food (e.g., 60% *tetraselmis* sp. should have a higher ingestion rate compared to 20% *tetraselmis* sp. because there is more non-toxic food in the former). This was not observed. Avoidance also cannot explain the evolutionary change observed when copepods were fed a non-limiting ration of non-toxic food supplemented with toxic *Alexandrium* spp. cells [17, 51].

While avoidance is not observed, preference for non-toxic over toxic cells can occur [52–54]. Some estimates indicate very high selective feeding, upwards of 90% against toxic cells when offered a choice of toxic and non-toxic food [52, 53, 55]. Selection of the magnitude may compromise the experimental design; however, using a similar experimental design to our own (i.e. a mixture of toxic and non-toxic *Alexandrium* spp.; including the same seed culture stock and source population of copepods) Senft et al. [56] found, at most, 35% bias against toxic cells. Since the current ingestion rates are for total *Alexandrium* spp. cells consumed, preference could not be tested, but food bias could have occurred in our experiments. If so, then this would serve to reduce the resolution of the toxicity gradient, but not invalidate the design. For example, under preference for non-toxic cells, the zero, 0.2, and 2.0 toxic cells ml^-1^ treatments in the current manuscript may have similar realized ingestion rates of toxic cells close to zero. These three treatments, however, would still be different than the higher toxic cell treatments, 20 & 200 toxic cells ml^-1^. This design would still test our hypotheses using three levels of toxicity: approximately zero, moderate, and high (sole food) toxicities. Regardless, if minor negative selection is enough to render the impact of the mutant isoform negligible, then this further supports the current findings that the mutant isoform is not the dominant factor in PST-adaptation.

### Costs

Costs of adaptation are assessed by measuring components of fitness in the absence of the stressor. There is conflicting information regarding costs associated with adaptation. Sodium channel mutations similar to that found in *Acartia hudsonica* may cause diseases in humans [24–28], indicating a potential cost of the copepod sodium channel mutation. Adaptations to metal toxicity come at a cost, typically in reduced overall fitness (lambda) or ability to withstand other stressors (e.g., temperature or salinity) in *Daphnia* spp. [57, 58] and the tide pool copepod *Tigriopus japonicus* [59]. Conversely, adaptation to stressors may not be costly. Ingestion of toxic cyanobacteria by *Daphnia* spp. had no evident cost [60–62]. Importantly, for our model copepod-toxic alga system, there was no difference in ingestion, egg production rate, and lambda on non-toxic food for PST-susceptible and tolerant *A*. *hudsonica* populations [17, 18]; this suggests little or no cost to PST-adaption. In our study, there was contradictory evidence for the role of the mutant sodium isoform in exacting a cost to individual *A*. *hudsonica*. There were no significant differences in EPR among expression groups when toxins were absent for both the toxin-dependent EPR (Figs 2 and 7A) and the reduction of EPR (Fig 8) experiments. Conversely, there was no relationship between I rate and EPR for the PMI group (Fig 5B). That is, higher ingestion rate did not necessarily result in increased EPR for the PMI expression group, which is evidence of a cost. Such a cost should be interpreted cautiously; it may not negatively affect the organism, or become a target of selection, because there was little difference in EPR between the PMI and PWI expression groups (Fig 2).

### Trade-offs

Cost and trade-off are often used interchangeably, implying they represent the same effect. We, however, restrict trade-off to represent a decrease in fitness of a genotype or phenotype across an environmental gradient. For example, *Fundulus heteroclitus* fish from polluted waters performed better in toxic sediments compared to non-toxic control conditions [63]. The authors refer to this as a cost of resistance, whereas we argue this is actually a trade-off since they are comparing the purported adapted population across an environmental gradient. Likewise, cadmium-resistant *Drosophila* lines showed reduced fecundity and emergence weight in cadmium-free environments compared to contaminated conditions [3]. We hypothesized that the PMI and EI groups would experience a trade-off, or reduction in a component of fitness, from toxic to non-toxic environments. This was only supported for GGE (Fig 4: bottom row). Lower toxic cell concentrations were associated with reduced GGE; however, there is no overall difference in GGE among the isoform expression groups, as described above. Thus, no ecological or evolutionary effects would result. There is no clear trade-off associated with the mutant sodium channel isoform.

### Interaction Effect between Toxin Dose and Isoform Expression Groups:

Significant interaction effects indicate the measurement of fitness (e.g., I, EPR, GGE) is dependent upon level of toxicity and expression group [64]. While the interaction effects suggest support for our central hypothesis, there is no consistent phenotypic evidence to suggest the mutant isoform is the dominant mechanism of adaptation. This interaction may indicate that the mutant isoform modifies the realized phenotypic expression of other genes or traits, but is not the dominant phenotypic determinant. Individual variability in selective and sloppy feeding, metabolism, and detoxification will all contribute to the interaction between sodium channel isoforms and toxin dose. Five different phenotypes of *Acartia hudsonica* with varying degrees of PST-tolerance were observed, suggesting that adaptation is controlled by a simple genetic system [21, 34]. The results of this study, however, seem to indicate that expression of the mutant sodium channel isoform does not appear to be the genetic system primarily contributing to adaptation. Taken together, there is no consistent evidence for a clear association between the relative proportion of mutant and wild-type sodium channel isoforms and adaptation to PSTs in *A*. *hudsonica*.

### Other Potential Mechanisms of PST-Adaptation in *Acartia hudsonica*

The IC_50_, STX concentration where 50% of the mutant sodium channels become blocked, is at ~1.6±0.14 x 10^-9^M STX [65]. Conservative calculations using copepod ingestion rates from the current dose-dependent experiments (Fig 1), *Alexandrium fundyense* cell toxicity (see methods), a gut volume of 10% of prosome volume, and toxin absorption efficiency of 10% day^-1^ [33] yield STX concentrations in the gut of ~ 4 x10^-6^–10^−9^ M. These are idealized calculations that do not include the binding affinity of the various PST congeners, loss due to sloppy feeding, or biochemical interactions within the gut. While more toxic strains of *Alexandrium* sp. have been recorded, the toxicity of the strain used here is within ranges repeatedly used by other researchers [16–18] and observed in nature. Further, induction from copepod feeding could have increased the cellular toxicity within each treatment [56], affecting the realized concentration experienced by each copepod; however, it is unknown how much induction took place. Overall, though, since any advantage of the mutation will be realized at intermediate concentrations (see discussion above), these calculations demonstrate the experimental design was appropriate to test our hypotheses.

Toxic cells that are ingested by a copepod interact first with cells lining the gut. It is unknown if, and at what rate, the PSTs are distributed throughout the rest of copepod body. For simplicity, we assumed that only the cells of a copepod gut are in direct contact with PSTs. Numerous voltage-gated sodium channel isoforms were found for the copepod *Calanus finmarchicus* [66]. In vertebrates, various sodium channel isoforms are expressed in different tissues [5, 26]. While only two isoforms were found in *Acartia hudsonica*, if only wild-type sodium channel isoforms are expressed in gut cells, then the advantage of the mutant isoform, if present, may never be realized.

Adaptation at the molecular level can be accomplished by four means: 1) constitutive over-expression of a gene, 2) constitutive under-expression of a gene, 3) structural changes in an affected gene, and 4) inducible expression of non-sensitive or compensatory genes [67, 68]. We found little evidence to support the hypothesis that a structural change in the affected gene (sodium channel) led to adaptation (mechanism 3 above). Overproduction of sodium channels is an alternative mechanism of adaptation to PSTs. That is, the number of sodium channels per cell is greater in adapted compared to susceptible individuals. Increased channels would create additional binding sites for toxins, effectively diluting the effect of a specific dose of toxin. The approach used in this study of measuring proportions of wild-type: mutant channels will not capture these differences. Quantifying the number of channels was beyond the scope of this study, but should be explored.

There are other ion channels that are susceptible to PSTs. Animals cells also contain non voltage-gated sodium channels, termed sodium leak channels, that aid in the creation of membrane charges and are susceptible to PSTs [69]. Saxitoxins can also bind to calcium channels [4]. Mutations, or differences, in these channels may work in independently, or in conjunction with the current sodium channel mutation, to confer tolerance. Tolerance may also involve de-toxification mechanisms. Copepods from exposed locations (Maine) ingested more toxic cells compared to naïve populations (New Jersey), but there was no difference in toxin accumulation [33]. Thus, the exposed copepods had lower toxin retention efficiencies; however, the mechanism of degradation was unknown. Lastly, proteins that neutralize PSTs [70–72], such as saxiphilin [70], may prevent toxins from binding with the sodium channel. The presence of these compounds remains largely unexplored in copepods, but deserves attention.
